# Influence of Chlorinating Agents on the Formation of Stable Biomarkers in Hair for the Retrospective Verification of Exposure

**DOI:** 10.1021/acs.analchem.2c01867

**Published:** 2022-11-22

**Authors:** Severin V. Martz, Matthias Wittwer, Chia-Wei Tan-Lin, Christian G. Bochet, Maximilian Brackmann, Christophe Curty

**Affiliations:** † Chemistry Division, Federal Office for Civil Protection, Spiez Laboratory, 3700 Spiez, Switzerland; ‡ Department of Chemistry, University of Fribourg, 1700 Fribourg, Switzerland; § Biology Division, Federal Office for Civil Protection, Spiez Laboratory, 3700 Spiez, Switzerland; ∥ Functional Genomics Center Zurich, University & ETH Zurich, 8057 Zürich, Switzerland

## Abstract

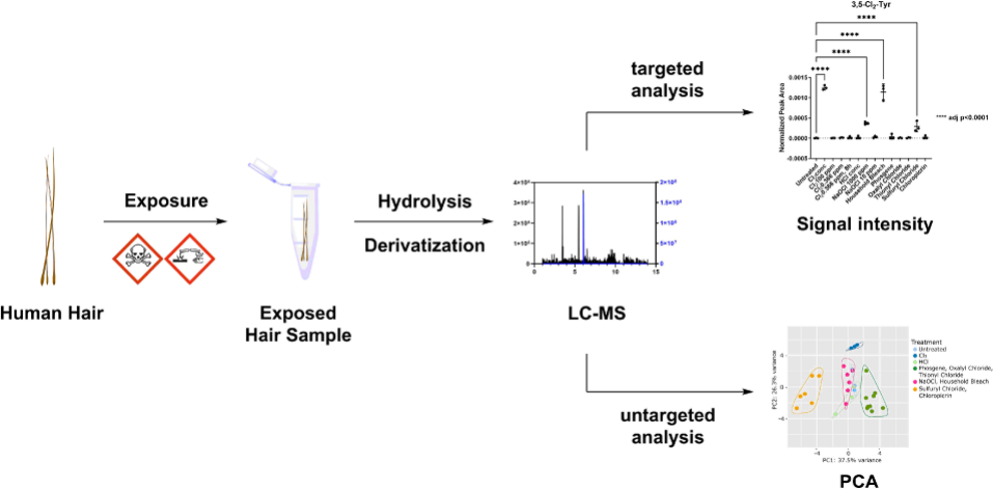

Chlorine, as a dual-use chemical, is an essential industrial chemical which has been used as a chemical weapon in the past due to its toxicity and availability. The retrospective verification of chlorine intoxication is often especially challenging, and unambiguous markers are still missing. In this study, the effects of different chlorinating and oxidizing agents on human hair were investigated. Samples were exposed to a variety of chlorinating chemicals for a short time and then completely hydrolyzed by a HBr solution to break down their keratin proteins into individual amino acids. After derivatization and targeted liquid chromatography-mass spectrometry analysis, 3-chlorotyrosine and 3,5-dichlorotyrosine were unambiguously identified from human hair exposed to chlorine, hypochlorite, and sulfuryl chloride. Our results show long-term stability of these markers in the biological matrix, as the chlorotyrosines can still be found 10 months post-exposure at the same levels. Finally, an untargeted analysis was able to discriminate between some of the different intoxicants.

## Introduction

Chlorine (Cl_2_) is a widely used chemical in industrial applications and is produced on a multi-ton scale worldwide. It plays a key role in water disinfection, polyvinyl chloride production, paper fabrication, and manufacturing of pharmaceuticals.^1−3^ However, Cl_2_ became also well-known as a chemical warfare agent (CWA) during World War I, where its first large-scale use was reported on April 22, 1915, when more than 150 tons of Cl_2_ were released, causing thousands of injuries and deaths.^4,5^ More recently, industrial accidents have caused injuries and death, for example, the train accident in Graniteville, South Carolina, in 2005.^1^ Cl_2_ has had its comeback as a chemical weapon in Iraq in 2006 and 2007, where the toxic gas had been used in terrorist attacks with suicide bombing trucks.^6−8^ The use of Cl_2_ on the modern battlefield in Syria was recently reported by the UN-supported Joint Investigative Mechanism (JIM) and by the OPCW Investigation and Identification Team (IIT) and the fact-finding mission (FFM).^9−13^ Dropped from helicopters as barrel bombs, the toxic chemical was dispersed over large areas affecting numerous persons. Moreover, Schneider and Lütkefend assessed that 336 chemical weapon attacks were credibly substantiated, confirmed, or comprehensively confirmed.^14^ Of these attacks, 89% were attributed to the use of Cl_2_. The attacks resulted in over 180 direct fatalities, and over 5000 people needed medical treatment after having been intoxicated.^14^ Although Cl_2_ is not listed in the Annex on Chemicals of the Chemical Weapons Convention (CWC), its use as a chemical weapon is prohibited under the CWC.^15^

Since Cl_2_ is a gas at ambient temperature, the main pathway of exposure occurs via inhalation, causing a variety of pulmonary effects.^2,16^ Cl_2_ reacts with moisture in the lungs and forms other reactive species, for example, hydrochloric acid and hypochlorous acid. These irritant and oxidative species cause damage to the airway tissues, resulting in decreased oxygen take-up and potentially suffocation.^2^ The threshold limit value is at 0.5 ppm, while a concentration of 1 to 3 ppm causes mild irritation of the mucus membranes.^2,17^ The onset of the pulmonary symptoms is at around 15 ppm. Concentrations higher than 400 ppm are supposed to be fatal within 30 min.^1^ Currently, Cl_2_ intoxication can only be treated supportively.^18^ Humidified oxygen has a positive effect on the victims until the proper oxygenation can be restored, and the inhalation of β-adrenergic agents helps against wheezing and coughing and supports normal breathing.^19,20^

Confirming the use of Cl_2_ as a CWA after a military or a terrorist attack is highly challenging. Hours after such an incident, Cl_2_ will have evaporated, and, lacking specific markers, its former presence can only be substantiated either by finding debris of special Cl_2_ release systems or based on the symptoms of victims in the aftermath of an attack.^14^ However, unequivocal confirmation of Cl_2_ exposure demands knowing and finding specific, easily accessible medical samples containing specific markers formed by the action of Cl_2_ in the human body.

**Figure 1:**
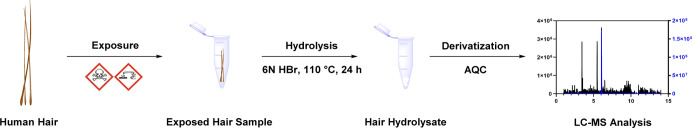
Workflow of this study. Human hair samples were exposed to the different chlorinating agents, followed by complete hydrolysis using 6 N HBr at 110 °C for 24 h. The obtained hydrolysate was derivatized with AQC and analyzed by LC–MS.

Earlier studies found chlorotyrosines, more specifically 3-chlorotyrosine and 3,5-dichlorotyrosine, in nasal tissue and blood plasma of rats after Cl_2_ exposure.^21,22^ These tyrosine derivatives have subsequently also been found in human blood, serum, and plasma when these biological matrices were exposed to Cl_2_.^23,24^ Animal studies have found other potential Cl_2_ biomarkers. 8-Isoprostane was found in the airways and blood of mice,^25^ while 2-chloropalmitaldehyde, 2-chloro-stearaldehyde (plus their oxidized products), 2-chloropalmitic acid (as a free acid and as esters), and 2-chloro-stearic acid were detected in the lungs and blood plasma of Cl_2_-exposed mouse and rat models.^26^l-α-Phosphatidylglycerol chlorohydrins were found in the bronchoalveolar lavage fluid (BALF) of mice exposed to the toxic gas.^27^ In an *in vivo* study with mice, Pantazides et al. showed that both chlorotyrosines can be detected in blood and lung samples for up to 24 h and in hair samples (whiskers) for up to 30 days post-chlorine exposure.^28^ Recently, Åstot and co-workers presented an optimized protocol for the detection of phospholipid chlorohydrin found in nasal lavage fluid (NLF) and BALF of rats exposed to Cl_2_. The biomarkers were found 24 h or 48 h after the exposure in NLF and BALF, respectively.^29^ Toprak et al. developed an approach for the diagnosis of Cl_2_ exposure based on Raman and Fourier-transform infrared (FTIR) spectroscopy for human nail samples.^30^ In this paper, we describe a straightforward and reproducible method to detect Cl_2_ intoxication from human hair with minimal sample preparation steps (Figure 1).

As chlorination is not necessarily caused by a reaction with Cl_2_, we also report the selectivity of different chlorinating agents for the formation of chlorotyrosines. Since Cl_2_ reacts to hydrochloric acid in the presence of moisture, this corrosive gas was included in the sample set. In addition, sodium hypochlorite (NaOCl) was chosen because it is an important industrial and household chemical. Phosgene and chloropicrin were chosen, as they have been used as CWAs in the past and are known for their high toxicity and their potential for chlorination.^16^ Oxalyl chloride, thionyl chloride, and sulfuryl chloride completed our sample set as these are widely used in industrial processes as chlorinating agents.^31−33^

Finally, we applied an untargeted analysis to identify secondary markers, when one marker is insufficient for unambiguous identification and, by this to discriminate between some of the different intoxicants.

## Experimental Section

### Materials

An analytical-grade AccQ-Tag kit [containing acetonitrile, borate buffer, and the 6-aminoquinolyl-*N*-hydroxysuccinimidyl carbamate (AQC) reagent] and Pierce Amino Acid Standard H (2.5 mM amino acid and 1.25 mM cystine) were purchased from Waters (Milford, MA, USA). A ^13^C-^15^N-labeled amino acid mixture, Metabolomics Amino Acid Mix Standard (MSK-A2), was obtained from Cambridge Isotope Laboratories (Tewksbury, MA, USA). Liquid chromatography (LC)-grade solvent was obtained from Biosolve (Dieuze, France).

### Hair Samples

Hair samples (brown hair) were collected from three healthy volunteers and were cut in 10 cm pieces for the exposure experiments. The hair samples were stored at room temperature. The collected hair samples were not bleached, frosted, or artificially colored.

### Chemicals

Pure Cl_2_, sulfuryl chloride, thionyl chloride, oxalyl chloride, and NaOCl solution (10–15%) were purchased from Sigma-Aldrich. Certified diluted Cl_2_ in N_2_ was purchased from Messer Schweiz AG. HCl gas was synthesized from CaCl_2_ and HCl 32% (Merck KGaA, Darmstadt, Germany), according to a protocol by Arnáiz.^34^ Household bleach was purchased as Cillit Bang Kraftreiniger Schimmel & Hygiene Duo. Diluted NaOCl was obtained by dilution with high-performance LC (HPLC)-grade H_2_O from J.T. Baker. Phosgene and chloropicrin were provided by Spiez Laboratory. The amino acid reference standards were purchased as follows: 3-chlorotyrosine, cysteic acid, methionine sulfoxide, and methionine sulfone were purchased from Sigma-Aldrich, and 3,5-dichlorotyrosine was purchased from abcr GmbH.

The exposure experiments were conducted under the adequate safety standards. The detailed exposure conditions are given in the Supporting Information (Table S1). All exposure experiments were carried out at room temperature with an exposure time of 10 min (except for one low Cl_2_ exposure carried out for 8 h at room temperature). Human hair samples (in biological triplicate) were exposed to the different chemicals under the conditions given in Table S1. The concentration given for bleach samples (NaOCl and household bleach) is the amount of “available chlorine”, which stands for hypochlorous acid and/or hypochlorite.^35^

### Sample Preparation

Hair samples were hydrolyzed to the level of individual amino acids using hydrolysis under acidic conditions at high temperature. To prevent the possible formation of any chlorinated artifacts during standard hydrolysis with hydrochloric acid, hydrobromic acid was used. Hair samples (50 to 100 μg) were hydrolyzed in 50 μL of 6 N HBr under 0.1% w/v phenol stream at 110 °C for 24 h and dried using SpeedVac.^36^ The samples obtained were derivatized with AQC based on the work of Cohen and the recommendations from the supplier (Waters).^37^ The derivatization mixture consisted of 25 μL of borate buffer, 5 μL of 500 μM MSK-A2 (Cambridge Isotope Laboratories), 10 μL of sample hydrolysate, and 10 μL of AQC solution. The solution was mixed thoroughly giving a total volume of 50 μL. The amino acids tryptophan, asparagine, and glutamine were not measured, as they are unstable during acid hydrolysis.^38^

### Amino Acid Analysis

Biological triplicates were analyzed on a Waters UPLC H-Class Plus system with an Acquity UPLC quadruple solvent manager and sample manager. Derivatized amino acids were detected on a Waters QDa single quadrupole mass detector in the positive ionization mode. Of each sample, 1 μL of derivatized amino acids was loaded on a Cortecs UPLC C18 1.6 μm column (2.1 × 150 mm, Waters, Cat. no. 186007096) maintained at 55 °C. Gradient elution was performed using 0.1% formic acid in water as eluent A and 0.1% formic acid in acetonitrile as eluent B. The flow rate was kept constant at 0.5 mL/min with the following gradient (expressed as solvent B): initial conditions: 1% B, 1.3 min: 1% B, 4.3 min: 13% B, 8.8 min: 15% B, 9.8 min: 95% B, 11.8 min: 95% B, 15 min: 1% B. Data was acquired with MassLynx 4.2 (Waters). The amino acids that served as reference chemicals (Figure S2) were derivatized and analyzed analogously to the hair samples. Their stability during sample hydrolysis was tested, and no change between hydrolyzed and non-hydrolyzed amino acids was found.

### Data Processing

Raw LC-mass spectrometry (MS) data sets were converted to mzXML files using MSConvert and subsequently retention time-aligned using the package xcms in RStudio.^39−41^ Peak areas of the compounds of interest were normalized to unmodified amino acids (Ala, Gly, Ile, Leu, Pro, and Val) in the same sample to account for differences in sample loading using Skyline.^42^ The peaks were automatically picked by the peak picking algorithm and manually checked and corrected if necessary. Extracted ion chromatograms were generated for the masses of interest, and integrated peak areas were exported from Skyline and analyzed in GraphPad Prism using analysis of variance (ANOVA) with the Bonferroni correction. The calculated adjusted p-values account for the Bonferroni correction of multiple testing.

### Untargeted Data Analysis

Retention time-aligned mzXML files were grouped by their treatment, centroided, and peak picked using a matched filter algorithm (xcms). Peak intensities were log2-transformed, and subsequently, peaks were grouped between the samples, and where necessary (i.e., missing peaks), intensity values were taken from raw data. Features (peaks) were ranked using shrinkage discriminant analysis (SDA). In the first step, features are selected which are statistically significantly over- or underrepresented between the treatment groups and ranked according to their correlation-adjusted *t*-score. In the second step, these features were used in principal component analysis (PCA). Two different rankings were performed, one to differentiate untreated, Cl_2_ conc-, HCl-, NaOCl-, household bleach- (1000 ppm), phosgene-, oxalyl chloride-, thionyl chloride-, sulfuryl chloride-, and chloropicrin-treated samples from each other and another grouped according to the presence of two identified markers, and NaOCl and household bleach treatments were grouped together. The resulting features for each of the two sets were manually checked for validity in Skyline. For better comparability in graphics, the dimensions in Figure 8 and PC1 in Figure 11 were multiplied by −1. The R-Script is available on request.

## Results and Discussion

**Figure 2:**
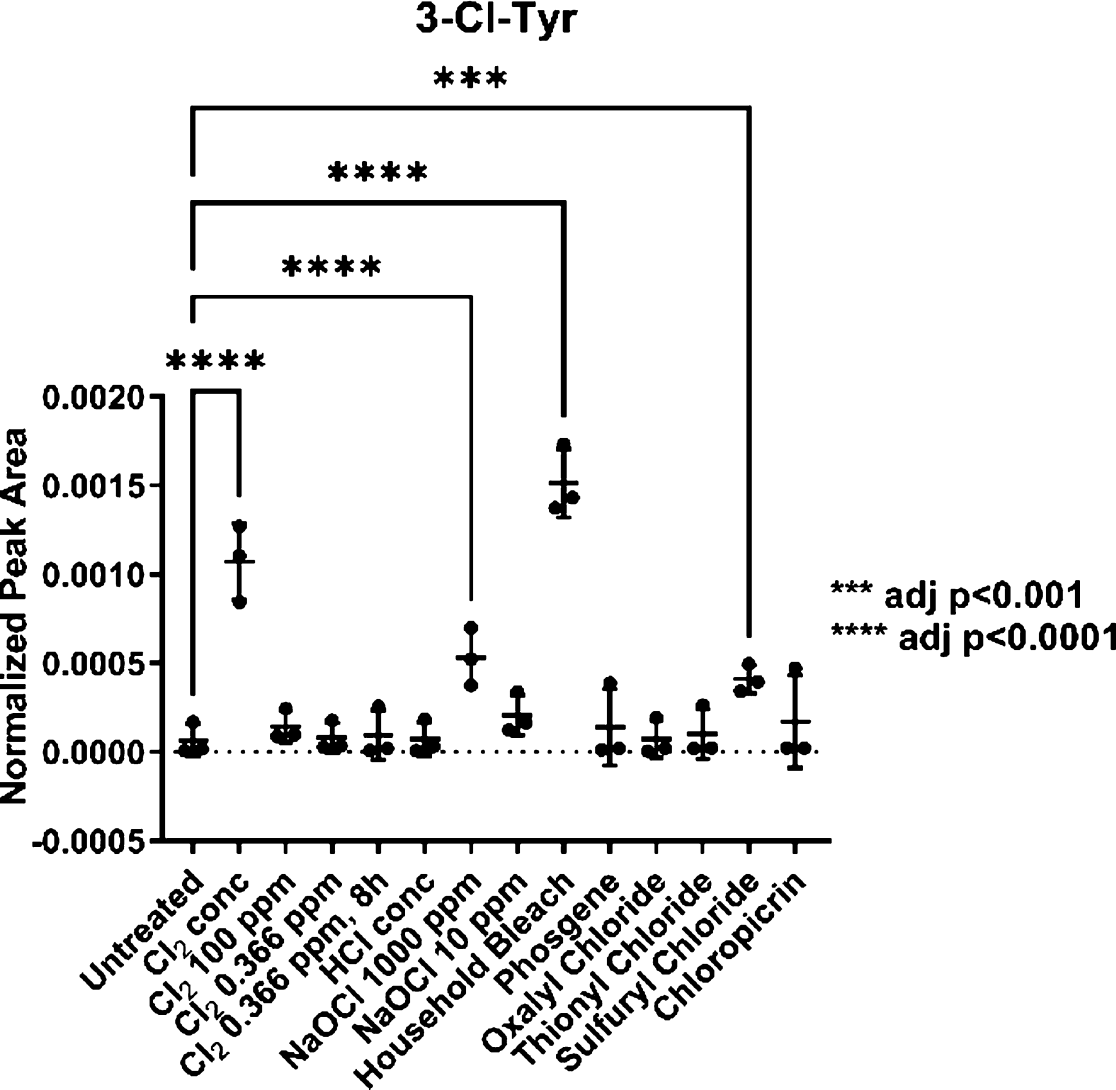
3-Chlorotyrosine (3-Cl-Tyr) is found in hair samples exposed to Cl_2_, NaOCl, household bleach, and sulfuryl chloride. All in biological triplicate. Exposure conditions are given in Table S1.

**Figure 3:**
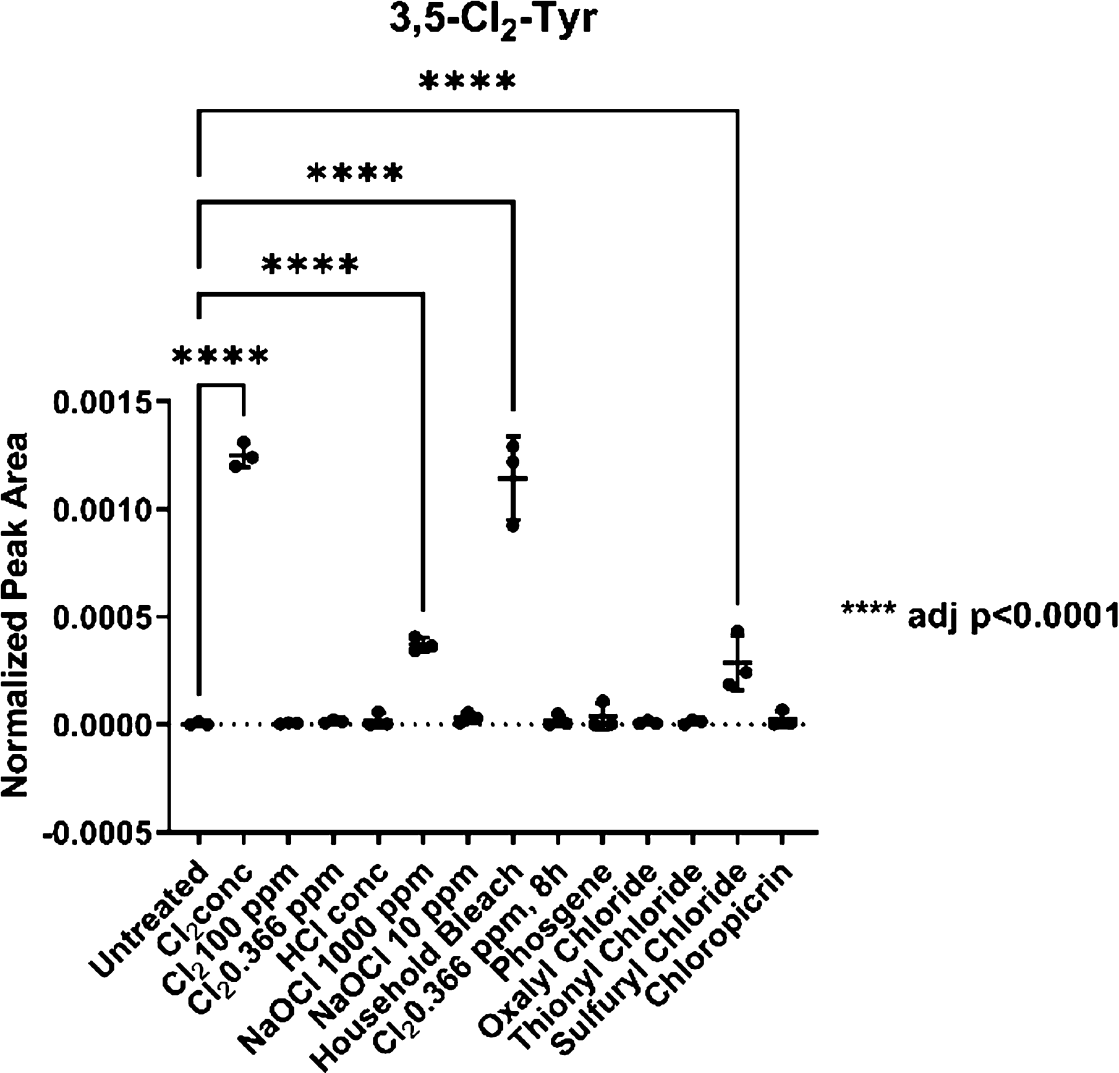
3,5-Dichlorotyrosine (3,5-Cl_2_-Tyr) is found in hair samples exposed to Cl_2_, NaOCl, household bleach, and sulfuryl chloride. All in biological triplicate. Exposure conditions are given in Table S1.

The phenolic side chain of tyrosine can undergo an electrophilic aromatic halogenation reaction upon treatment with certain chlorinating agents, such as Cl_2_, bleach, and/or sulfuryl chloride (Figures 2 and 3). We also found these modifications in human hair in significantly higher amounts when using concentrated Cl_2_, NaOCl (1000 ppm), household bleach, and sulfuryl chloride for the intoxication experiments, compared with those in the untreated control. These compounds were not observed in significant amounts when hair samples were not exposed to chlorinating agents or to lower concentrations of Cl_2_ (100 or 0.366 ppm) or NaOCl (10 ppm) or any other chlorinating or oxidizing agent selected for our study (Table S1).

The samples treated with pure Cl_2_, NaOCl (1000 ppm), and household bleach contained significant amounts of 3-chlorotyrosine and 3,5-dichlorotyrosine when compared to the untreated control (*p* < 0.0001). Additionally, the samples treated with sulfuryl chloride were also found to contain 3-chlorotyrosine and 3,5-dichlorotyrosine (*p* < 0.001) but at a lower level compared to the most reactive chemicals observed. The detection of chlorotyrosines in the samples of Cl_2_ and sulfuryl chloride should be compared with caution to that in the samples of household bleach and NaOCl (1000 ppm), as the exposure of the hair samples to household bleach and NaOCl (10 and 1000 ppm) was carried out in the liquid phase, whereas all other intoxication experiments were carried out in the gas phase. All of these chemicals seem to react with the electron-rich aromatic ring of the side chain of tyrosine presumably in an electrophilic aromatic substitution reaction in ortho positions to the hydroxyl group. The finding of the chlorotyrosine derivatives in bleach samples is consistent with results of other studies.^43,44^ Similarly, the detection of the chlorotyrosines in the sulfuryl chloride sample is in accordance with many literature protocols, where this chemical is used for electrophilic aromatic chlorination of tyrosine derivatives.^45,46^ Since the water of many swimming pools is disinfected using Cl_2_ or other chlorinating agents, hair samples of a frequent swimmer were additionally analyzed with the same method.^47^ The chlorotyrosines were not observed in a significant amount from the swimmer’s hair samples (Figure S11). The chlorine concentration therefore presumably appears to be too low to be able to form chlorotyrosines to any significant extent.

For practical applications in the field, a biomarker requires stability.^48^ We therefore tested whether the chlorotyrosines in the Cl_2_-exposed samples remained detectable in hair samples 2.5 and 10 months after exposure.

**Figure 4:**
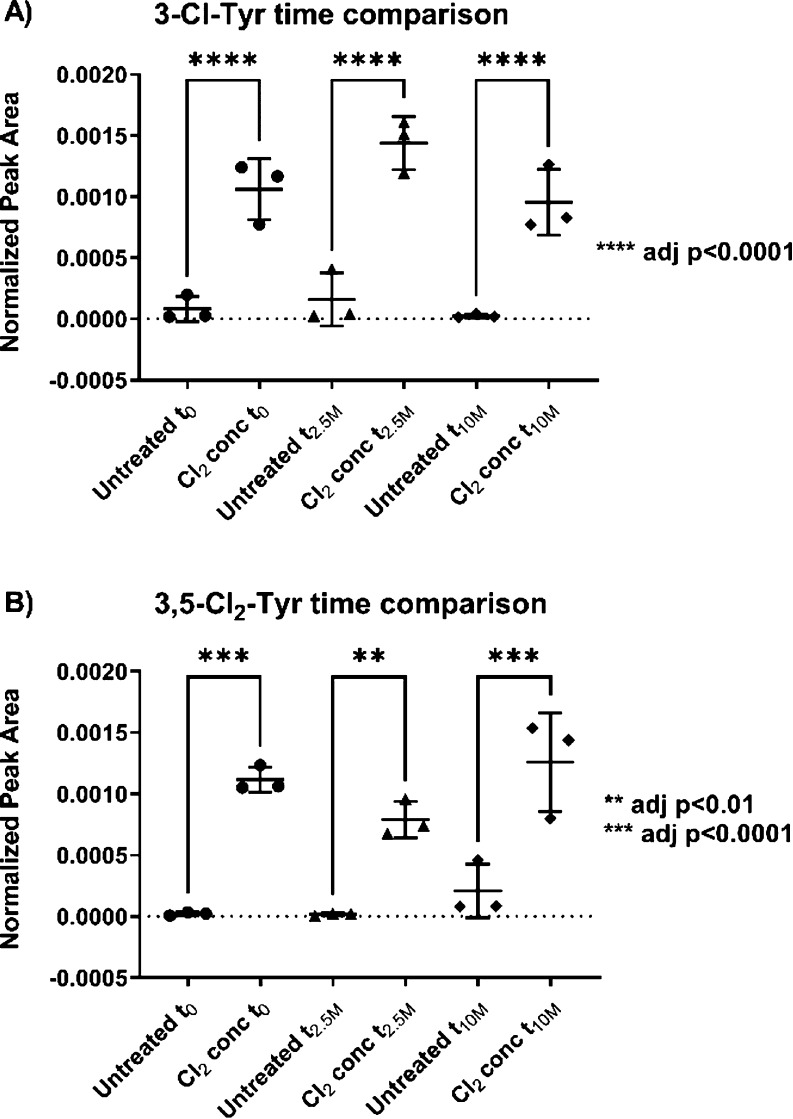
Chlorotyrosines are stable over extended time periods. (A) 3-Cl-Tyr and (B) 3,5-Cl_2_-Tyr are suitable markers for the long-term verification of exposure to certain chemicals.

We measured one part of the human hair samples exposed to Cl_2_ at *t*_0_, while other parts of the same sample were kept in an Eppendorf tube at room temperature for 2.5 or 10 months. After this time, the samples were processed and analyzed in the same way (*t*_2.5M_ and *t*_10M_). We were able to show that the results from the samples measured 2.5 or 10 months after exposure did not differ from those measured directly after exposure as the amounts of 3-chlorotyrosine and 3,5-dichlorotyrosine are comparable and still contain significantly more (*p* < 0.01) than the untreated control. Although dechlorination of chlorotyrosines *in vivo* is known, we could not observe a significant degradation of 3-chlorotyrosine and 3,5-dichlorotyrosine in human hair, meaning that the two types of chlorotyrosines show a high level of stability in this biological matrix (Figure 4).^49^ This is presumably because human hair is not metabolically active and can be considered more as an inert environmental sample. This finding might be highly relevant for authorities or international organizations that wish to apply chlorotyrosine as a biomarker to confirm Cl_2_ exposure of victims using hair as a sample.

**Figure 5:**
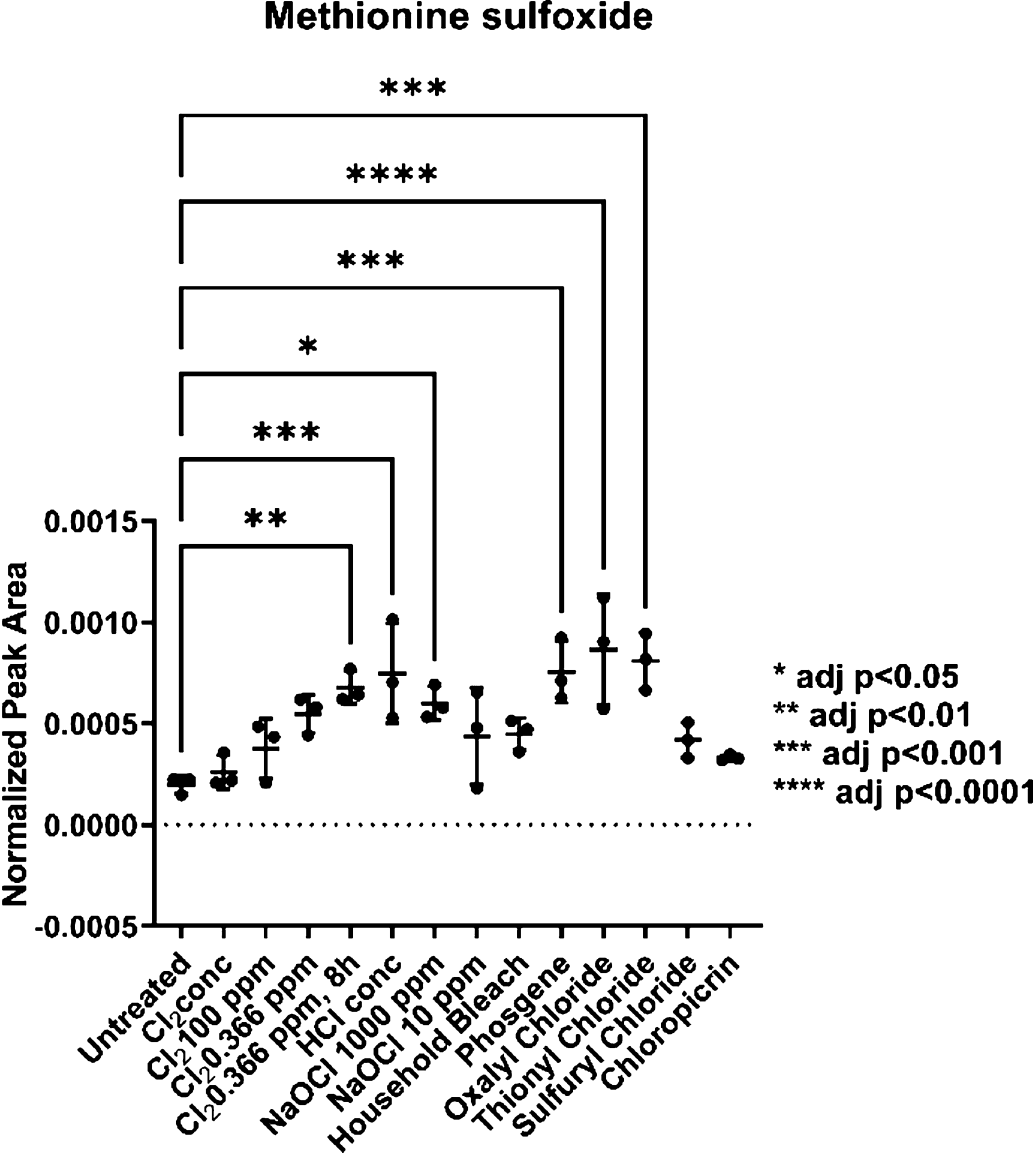
Methionine sulfoxide was found in all samples but in elevated amounts in the case of Cl_2_ (0.366 ppm for 8 h), HCl conc., NaOCl (1000 ppm), phosgene, oxalyl chloride, and thionyl chloride.

**Figure 6:**
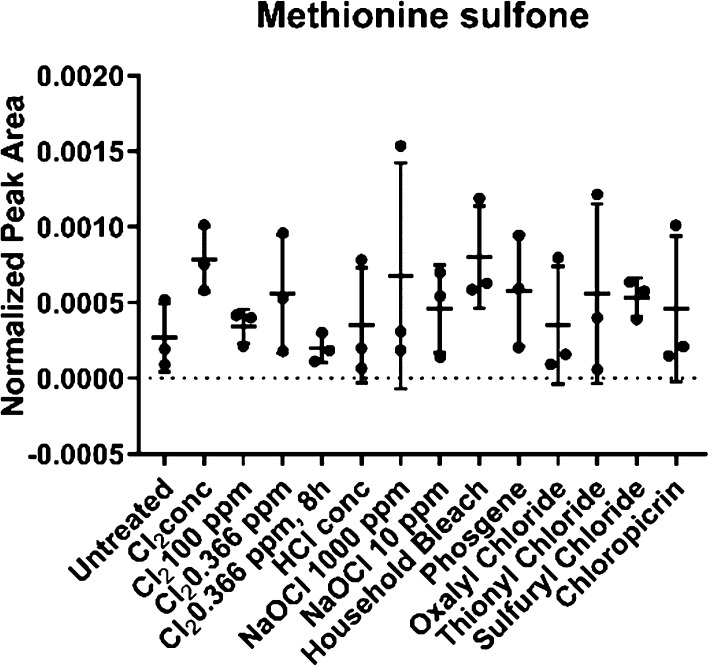
Methionine sulfone showed no significant differences between untreated and intoxicated hair.

Since nearly all of the chemicals in our study are not only chlorinating but also oxidizing species, we furthermore investigated specific oxidations on the easily oxidizable amino acid side chains of methionine and cysteine to test for other potential markers.^44^ The side chain of methionine can be oxidized to the sulfoxide species *in vitro* and *in vivo* by HOCl.^44,50^ We detected a background level of methionine sulfoxide (mono-oxidized methionine) through all treatments and in the untreated hair sample. However, an elevated amount of methionine sulfoxide was found in the sample exposed to Cl_2_ 0.366 ppm for 8 h (*p* < 0.01), HCl conc. (*p* < 0.01), NaOCl (1000 ppm) (*p* < 0.05), phosgene (*p* < 0.001), oxalyl chloride (*p* < 0.0001), and thionyl chloride (*p* < 0.001) (Figure 5). The samples treated with oxalyl chloride, thionyl chloride, and phosgene showed the highest significance. In samples intoxicated with concentrated Cl_2_ and also lower-concentrated (100 ppm), NaOCl 10 ppm, household bleach, and sulfuryl chloride, no significant increases could be observed. The finding of methionine sulfoxide through all samples suggests that methionine is easily oxidized to methionine sulfoxide, which is consistent with other studies. The methionine side chain is vulnerable to reactive oxygen species like HOCl or H_2_O_2_.^44,51^ However, the reaction is reversible *in vivo* as methionine sulfoxide reductase enzymes catalyze the back reaction to methionine.^51^ As these enzymes are not present in hair, the oxidation is assumed to be irreversible. Consequently, oxidation of methionine is not specific to Cl_2_. No significant differences between untreated and treated hair were found for methionine sulfone (Figure 6).

**Figure 7:**
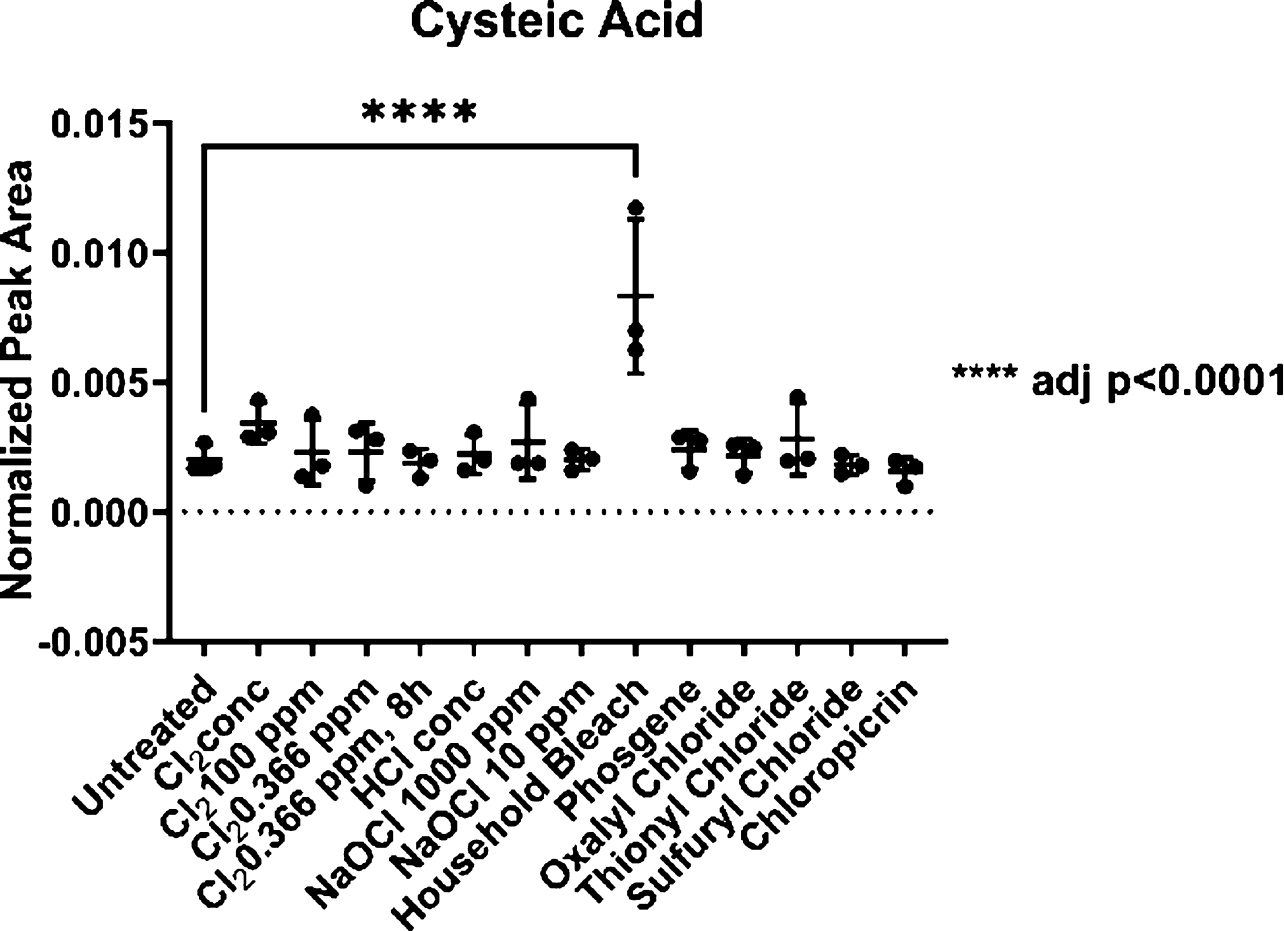
Cysteic acid is detectable in all samples at background levels and can be found in significantly higher amounts in the household bleach-treated hair compared to that in the untreated control.

Cysteine is known to undergo oxidation via sulfenic and sulfinic acid to sulfonic acid (cysteic acid).^52^ Our experiments showed that all samples contained a certain background level of cysteic acid, but only the treatment with household bleach produced significant levels (p > 0.0001) of cysteic acid (Figure 7). The background level of cysteic acid can be explained by the high sensitivity of cysteine to oxidizing chemicals and/or UV radiation.^53^ Various studies confirmed that in the case of hair bleaching, the amount of cysteine decreases, and the amount of cysteic acid increases.^54^

**Figure 8:**
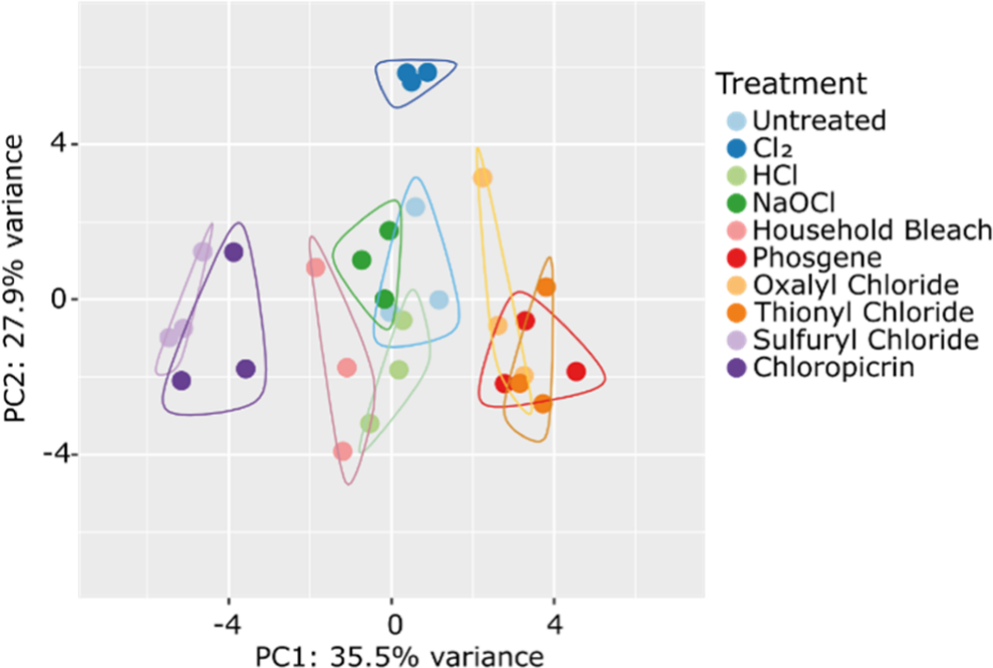
Some groups of treatments can be discerned by PCA. Cl_2_ conc. treatment is very distinct from all other treatments, while sulfuryl chloride and chloropicrin and phosgene, oxalyl chloride, and thionyl chloride form distinct groups in the PCA.

We found chlorinated tyrosines after treatment with Cl_2_, NaOCl (and household bleach), and sulfuryl chloride; however, as described above, an optimal marker would be specific for exactly one chemical. In the absence of one specific marker, one can in principle also use a combination of different markers, if these are sufficiently different between the respective samples. To identify a suitable combination of markers, we performed an untargeted analysis of the data to determine possible discriminatory masses in these data sets. Therefore, we grouped the data sets according to sample treatment and performed an SDA with subsequent PCA. SDA is a supervised learning algorithm which corrects for the influence of highly correlating features between the data sets to be compared (i.e., many more features than samples). This is important to avoid over-parametrization of the model. An untargeted approach should reflect at least the previously identified masses of the two chlorotyrosines (and their isotopes), and if there are any other relevant discriminatory features, these should also be identified. First, we compared many of the treatments [Cl_2_ conc, HCl, NaOCl, household bleach (1000 ppm), phosgene, oxalyl chloride, thionyl chloride, sulfuryl chloride, and chloropicrin] to each other (Figure 8) and could confirm the importance of chlorotyrosines for the differentiation. Furthermore, the PCA indicated that some features exist that allow the differentiation of more groups of treatments. Intoxication with Cl_2_ can clearly be differentiated from all other treatments. Treatment with NaOCl or household bleach is only discernible from the untreated control in the third dimension of the PCA (Figure S10).

**Figure 9:**
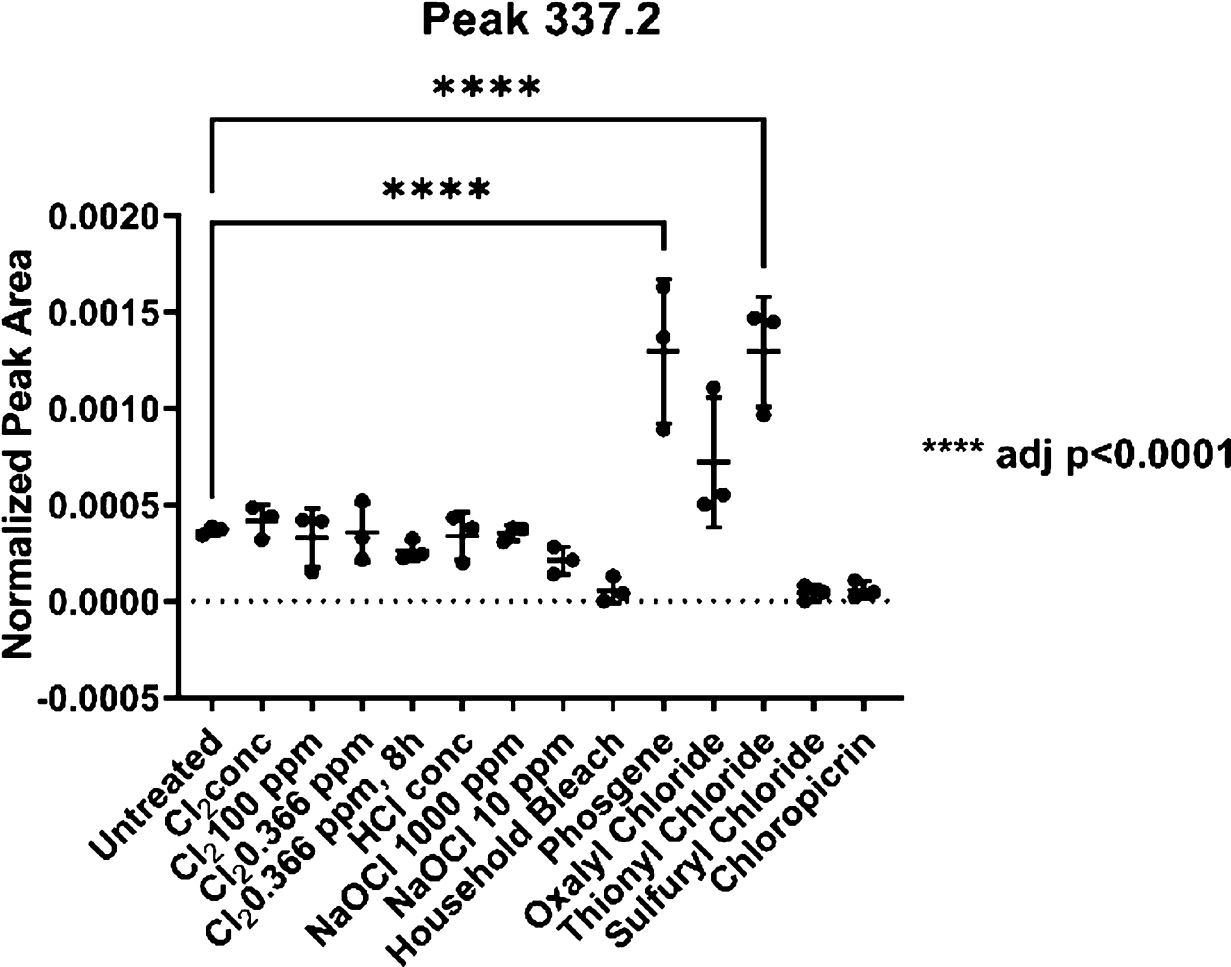
The untargeted approach identified two interesting features. A compound with an *m*/*z* of 337.2 was found in a significant amount in the phosgene- and thionyl chloride-treated samples.

**Figure 10:**
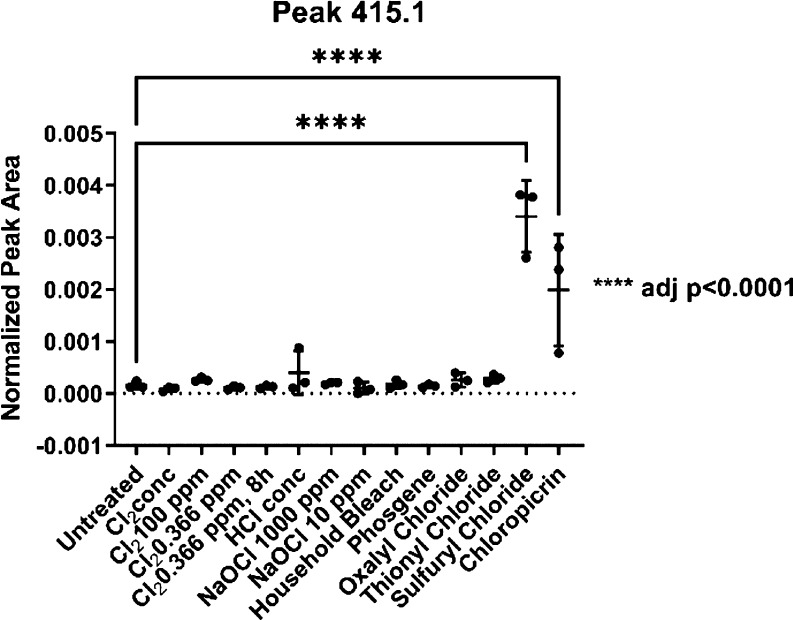
A compound with an *m*/*z* of 415.1 was found in significant amounts in the samples treated with sulfuryl chloride or chloropicrin.

We investigated the nature of these features further and manually inspected all relevant masses in Skyline. Thereby, we could confirm the importance of two more features, a feature of a mass-to-charge ratio of 337.2 *m*/*z* and another one of 415.1 *m*/*z* (Figures 9 and 10).

**Figure 11:**
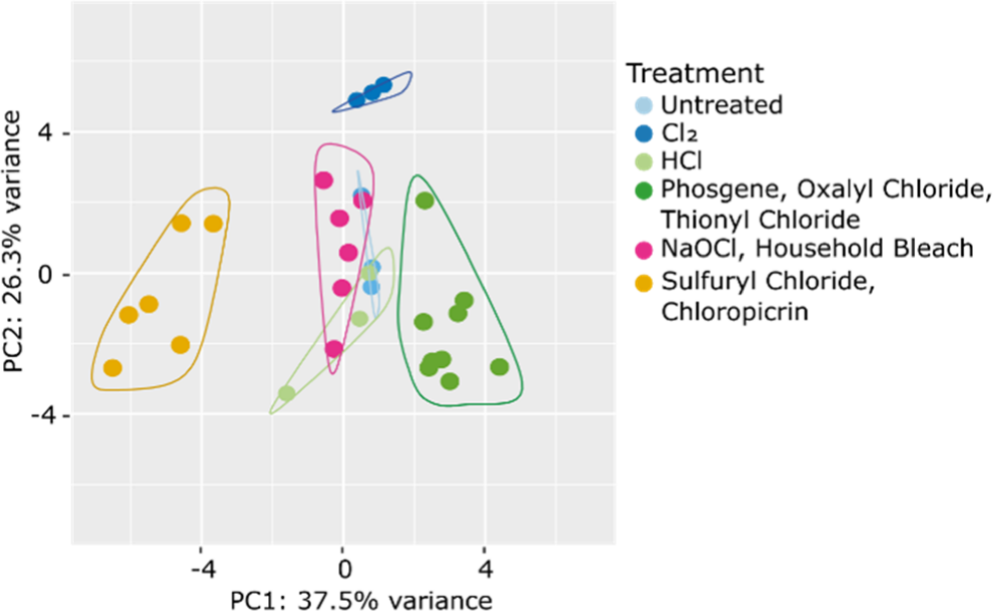
Groups of treatments can be distinguished by PCA. Grouping by the presence of *m*/*z* 337.2 (green) and *m*/*z* 415.1 (orange) results in better separation of the groups.

Grouping of those treatments that exhibited the same “new” features, that is, *m*/*z* 337.2 or *m*/*z* 415.1, and re-analysis by SDA and PCA resulted in readily distinguishable groups in the PCA (Figure 11).

A signal at 415.1 *m*/*z* can also be used to differentiate between exposure to sulfuryl chloride versus Cl_2_ conc., NaOCl, and household bleach. Furthermore, if this signal is present (415.1 *m*/*z*) but none of the two chlorotyrosines are, exposure to chloropicrin (*p* < 0.001) might be suspected (Figure 10).

In summary, the significant differences between intoxicated and untreated samples can be viewed in Table 1. Both chlorotyrosines were found in significantly higher amounts in hair exposed to Cl_2_ conc, NaOCl (1000 ppm), household bleach, and sulfuryl chloride, compared to those in the untreated hair. If further differentiation is needed, the features found in the untargeted approach can be used.

**Table 1:** Summary of all Conditions with Significant Discerning Peaks with “x”

	3-Cl-Tyr	3,5-Cl_2_-Tyr	337.2 *m*/*z*	415.1 *m*/*z*
Cl_2_ conc.	x	x		
NaOCl (1000 ppm)	x	x		
household bleach	x	x		
sulfuryl chloride	x	x		x
thionyl chloride			x	
phosgene			x	
chloropicrin				x

Although we found two additional discriminatory masses, the sum of many more features aids in separation of the groups in the PCA; however, these are not the single most important features and thus were not considered further.

## Conclusions

From a practical perspective, we established a simple method of sample collection, processing, and analysis, which could be of great importance for retrospective verification of Cl_2_ exposure. In this study, we have shown that human hair can be used as a biological matrix to detect Cl_2_ exposure. The detection of 3-chlorotyrosine and 3,5-dichlorotyrosine in human hair shows that Cl_2_ reacts with tyrosines in hair and leads to the formation of stable adducts. We showed that these adducts can still be detected 10 months after exposure. Furthermore, we have determined that these adducts can also be formed when exposing human hair to high bleach concentrations or sulfuryl chloride.

Chlorotyrosines are useful to detect exposure to Cl_2_ but cannot be used solely to differentiate between the many chlorinating chemicals that were used in this study. The focus in the search for Cl_2_ biomarkers should therefore not be exclusively on Cl_2_, as other chlorination reagents can also form chlorotyrosines.

We showed that the three species methionine sulfoxide, methionine sulfone, and cysteic acid were present in all hair samples, including the untreated one. Cysteic acid was only found in significantly higher amounts compared to that in the untreated control in the case of exposure to household bleach. Significant increase in the amounts of methionine sulfoxide was found in the case of NaOCl, oxalyl chloride, phosgene, and thionyl chloride exposure.

An untargeted analysis of the data obtained was performed using SDA and PCA, and further discriminatory masses were identified. In accordance with the targeted analysis, chlorotyrosines were found to be the main discerning features. Upon trying to further discriminate between the chlorinating agents, the two masses *m*/*z* 337.2 and *m*/*z* 415.1 were found. Considering the presence of a signal for chlorotyrosines, a signal at 415.1 *m*/*z* is indicative of sulfuryl chloride intoxication, while a signal at 415.1 *m*/*z* in the absence of chlorotyrosines suggests an intoxication with chloropicrin. Similarly, a signal at 337.2 *m*/*z* is indicative of thionyl chloride or phosgene intoxication.

## Supporting Information Available

Detailed exposure conditions, amino acid reference standards, principle of derivatization, extracted ion chromatograms of the reference material, total ion chromatogram of an untreated hair sample, separated PCA dimensions PC1 vs PC3, and chlorotyrosine analysis in swimmer’s hair samples (PDF)
