# Supplementary material for: Influence of Chlorinating Agents on the Formation of Stable Biomarkers in Hair for the Retrospective Verification of Exposure
Source: Anal Chem. 2022 Nov 22;94(48):16579–86. doi: 10.1021/acs.analchem.2c01867 (PMC9730300; doi:10.1021/acs.analchem.2c01867)
Supplement: Supplementary file 1 — ac2c01867_si_001.pdf [file ac2c01867_si_001.pdf]

Supporting Information:

Influence of Chlorinating Agents on the Formation of Stable Biomarkers in Hair for the Retrospective Verification of Exposure

Severin V. Martz[1,2], Matthias Wittwer[3], Chia-Wei Tan-Lin[4], Christian G. Bochet[2], Maximilian Brackmann[3*], Christophe Curty[1*]

[1] Spiez Laboratory, Chemistry Division, Federal Office for Civil Protection, 3700 Spiez, Switzerland

[2] University of Fribourg Department of Chemistry, 1700 Fribourg, Switzerland

[3] Spiez Laboratory, Biology Division, Federal Office for Civil Protection, 3700 Spiez, Switzerland

[4] Functional Genomics Center Zurich, University & ETH Zurich, 8057 Zürich, Switzerland

E-Mail: maximilian.brackmann@babs.admin.ch & christophe.curty@babs.admin.ch

Contents

Exposure experiments S-2

MS parameters S-5

Reference material & derivatization S-5

Extracted ion chromatograms of the reference material S-6

Exposure experiments

The exposures of hair samples to Cl_2, HCl, phosgene, chloropicrin, oxalyl chloride, sulfuryl chloride and thionyl chloride were carried with the chlorinating chemicals being in the gas phase. Whereas the exposure to NaOCl and household bleach was carried out with the chemicals being in an aqueous solution (Table S 1).

Table S 1. Chemicals used for exposure experiments under the given conditions.

**Chemical**	**Concentration/Amount**	**Structure**	**Conditions**
Chlorine	concentrated	Cl_2	r.t., 10 min. Gas exposure.
Chlorine in N_2	100 ppm ±3%	Cl_2	r.t., 10 min. Gas exposure in N_2
Chlorine in N_2	0.366 ppm ±10%	Cl_2	r.t., 10 minutes and 8 h. Gas exposure in N_2
HCl	10’000 ppm ±25%	HCl	r.t., 10 minutes. Gas exposure in air.
NaOCl	1000 ppm ±10%	NaOCl	r.t., 10 min. Aqueous exposure.
NaOCl	10 ppm ±10%	NaOCl	r.t., 10 min. Aqueous exposure.
Household bleach	<5% Cl-based bleaching agents	NaOCl	r.t., 10 min. Aqueous exposure.
Phosgene	≈2 mmol/L	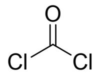	r.t., 10 minutes. Gas exposure in air.
Oxalyl chloride	≈2 mmol/L	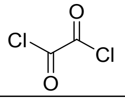	r.t., 10 minutes. Gas exposure in air.
Thionyl chloride	≈2 mmol/L	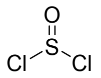	r.t., 10 minutes. Gas exposure in air.
Sulfuryl chloride	≈2 mmol/L	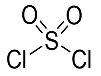	r.t., 10 minutes. Gas exposure in air.
Chloropicrin	≈2 mmol/L	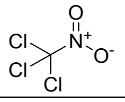	r.t., 10 minutes. Gas exposure in air.

Chlorine:

The exposure experiments were carried out in an apparatus consisting of the Cl_2 cylinder with a pressure reducing valve, a first gas washing bottle (250 mL), the reaction vessel (100 mL two-necked round-bottomed flask) and a second gas washing station containing an aqueous 10 % NaOH solution to neutralize the Cl_2.

The hair samples were placed in the reaction apparatus and Cl_2 (either concentrated, 100 ppm (+/- 3% relative) in N_2 or 0.366 ppm (+/- 10% relative) in N_2 were passed through the apparatus at room temperature for 10 minutes or 8 h, respectively. The remaining Cl_2 was removed by flushing with argon gas. The hair samples were collected and stored in Eppendorf tubes at room temperature under normal atmosphere (air).

NaOCl:

Hair samples were placed in aqueous NaOCl solution (1.5 mL) with concentrations of approx. 1’000 ppm ±10% and 10 ppm ±10% at room temperature for 10 minutes. The hair samples were collected and dried with a Kleenex. Upon drying at room temperature for 2 h the samples were stored in Eppendorf tubes at room temperature.

Household bleach:

Hair samples were placed in commercially available household bleach solution (1.5 mL) at room temperature for 10 minutes. The hair samples were collected and dried with a Kleenex. Upon drying at room temperature for 2 h the samples were stored in Eppendorf tubes at room temperature.

HCl:

HCl was synthesized following a protocol by Arnáiz et al.[34] $CaCl_2$ (25 g, 225 mmol) was added to a 250 mL three-necked round-bottomed flask equipped with addition funnel and a gas outlet tube connected to the reaction chamber, a 100 mL two-necked round-bottomed flask. The gas was trapped using two gas washing tubes with deionized H_2O (50 mL). The hair samples were placed into the reaction chamber and a solution of HCl (32%, 25 mL) was added dropwise over 10 minutes to the $CaCl_2$ forming white fumes. A constant stream of HCl could be observed in the gas washing tube. The hair samples were stored in Eppendorf tubes at room temperature.

Phosgene:

Phosgene (1 g, 10 mmol) was added to a 5’000 mL Schott flask. The flask was sealed with a stopper and Teflon tape. The flask was left stand at 19 ˚C for 19 h to let the phosgene evaporate. The phosgene did not evaporate completely. The flask was opened quickly, and the hair samples were placed according to Figure S 1. The hair samples were let in the flask for 10 minutes at 19 ˚C. The hair samples were removed and the upper part of the hair, which was not in contact with the vial (holder) was cut and transferred to Eppendorf tubes at room temperature.

Chloropicrin:

Chloropicrin (970 µL, 10 mmol) was added to a 5’000 mL Schott flask. The flask was sealed with a stopper and Teflon tape. The flask was left stand at 19 ˚C for 19 h to let the chloropicrin evaporate. The chloropicrin did not evaporate completely. The flask was opened quickly, and the hair samples were placed according to Figure S 1. The hair samples were let in the flask for 10 minutes at 19 ˚C. The hair samples were removed and the upper part of the hair, which was not in contact with the vial (holder) was cut and transferred to Eppendorf tubes at room temperature.

Oxalyl chloride:

Oxalyl chloride (858 µL, 10 mmol) was added to a 5’000 mL Schott flask. The flask was sealed with a stopper and Teflon tape. A white fog was formed at the bottom of the flask. It seemed that oxalyl chloride reacted with moisture in the flask and settled at the glass wall. After letting the liquid evaporate at 21 ˚C for 1 h, the flask was opened quickly, and the hair samples were placed according to Figure S 1. The hair samples were let in the flask for 10 minutes at 21 ˚C. The hair samples were removed and the upper part of the hair, which was not in contact with the vial (holder) was cut and transferred to Eppendorf tubes at room temperature.

Sulfuryl chloride:

Sulfuryl chloride (808 µL, 10 mmol) was added to a 5’000 mL Schott flask. The flask was sealed with a stopper and Teflon tape. After letting the liquid evaporate at 21 ˚C for 1 h, the flask was opened quickly, and the hair samples were placed according to Figure S 1. The hair samples were let in the flask for 10 minutes at 21 ˚C. The hair samples were removed and the upper part of the hair, which was not in contact with the vial (holder) was cut and transferred to Eppendorf tubes at room temperature.

Thionyl chloride:

Thionyl chloride (726 µL, 10 mmol) was added to a 5’000 mL Schott flask. The flask was sealed with a stopper and Teflon tape. After letting the liquid evaporate at 21 ˚C for 1 h, the flask was opened quickly, and the hair samples were placed according to Figure S 1. The hair samples were let in the flask for 10 minutes at 21 ˚C. The hair samples were removed and the upper part of the hair, which was not in contact with the vial (holder) was cut and transferred to Eppendorf tubes at room temperature.

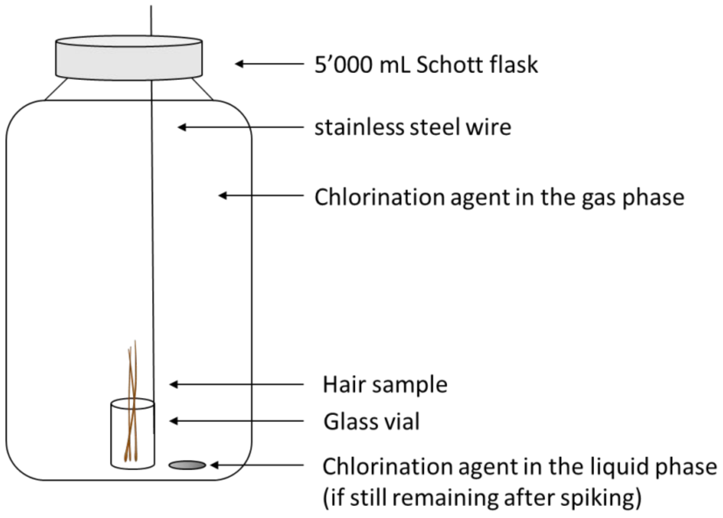

Figure S 1. Exposure setting for phosgene, thionyl chloride, oxalyl chloride, sulfuryl chloride and chloropicrin.

MS parameters

The single quadrupole mass spectrometer was used with the following parameters. The cone voltage was set to 10 V for all amino acids and the probe temperature was set to 600°C. The voltage of electrospray was set to 0.6 KV.

Reference material & derivatization

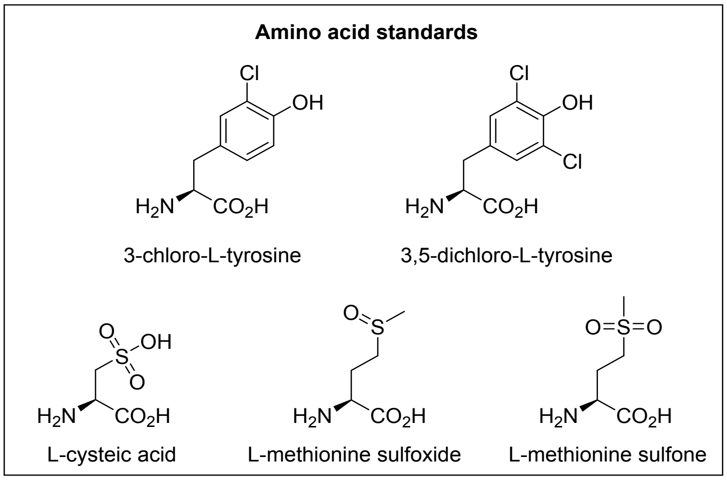

Figure S 2. Standards of amino acids used as reference material.

The hydrolysate of the hair samples, as well as the amino acid reference standards, were derivatized with AQC.[37] A reaction scheme is depicted in Figure S 3. The amino acids asparagine, glutamine and tryptophan were not measured since asparagine and glutamine are converted into aspartic acid and glutamic acid respectively under the strong acidic conditions. Tryptophan is prone to degradation under acidic conditions and was therefore also not measured.[38] The compounds of interest were verified by the analysis of reference material (Figure S 4 - 8).

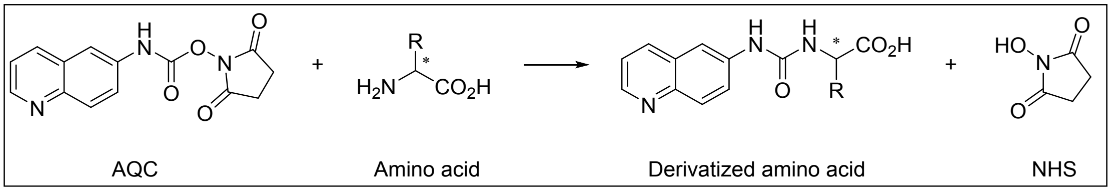

Figure S 3. Derivatization reaction using 6-aminoquinolyl-*N*-hydroxysuccinimidyl carbamate (AQC) that reacts with the free amino group of the amino acid to give the derivatized amino acid and *N*-hydroxysuccinimide (NHS).

Extracted ion chromatograms of the reference material

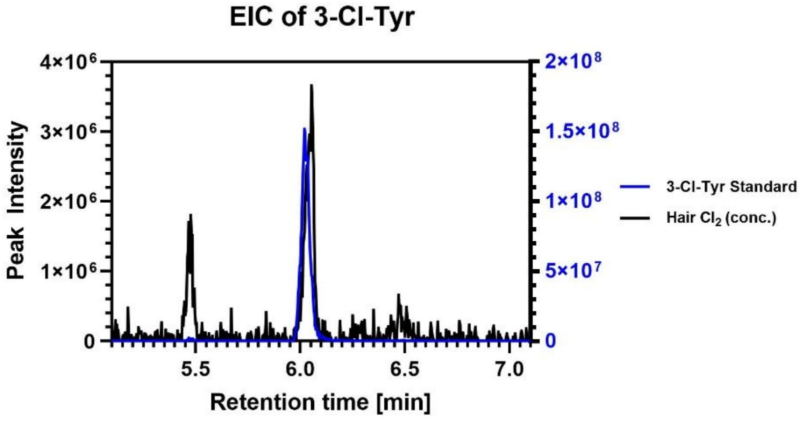

Figure S 4. Comparison of extracted ion chromatograms (EIC) of 3-chlorotyrosine (3-Cl-Tyr) obtained from human hair exposed to chlorine conc. (black) and the reference material (blue).

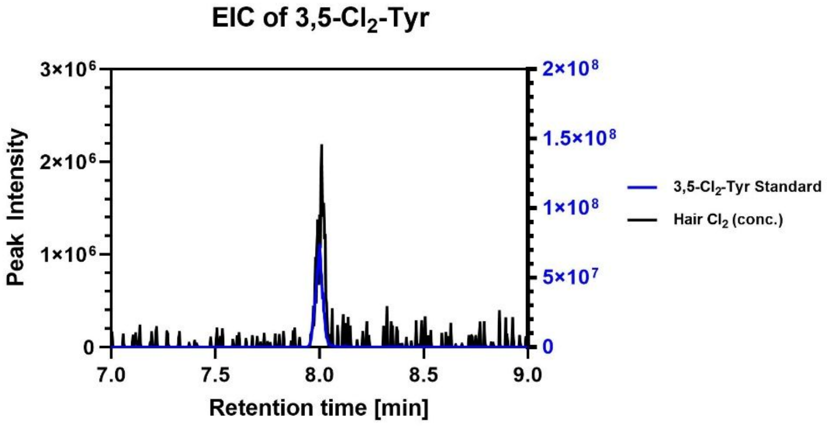

Figure S 5. Comparison of extracted ion chromatogram (EIC) of 3,5-dichlorotyrosine (3,5-Cl_2-Tyr) obtained from human hair exposed to chlorine conc. (black) and the reference material (blue).

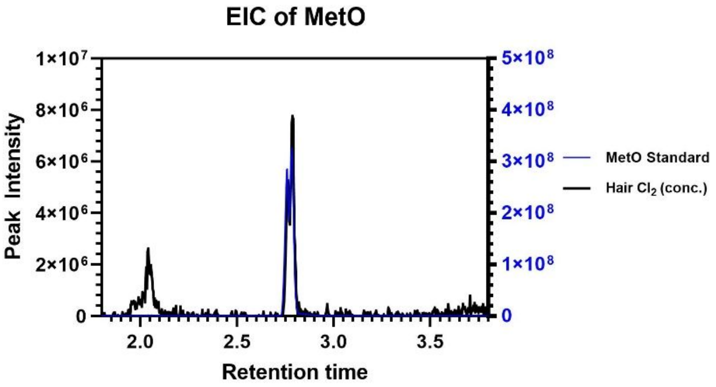

Figure S 6. Comparison of extracted ion chromatogram (EIC) of methionine sulfoxide (MetO) obtained from human hair exposed to chlorine conc. (black) and the reference material (blue).

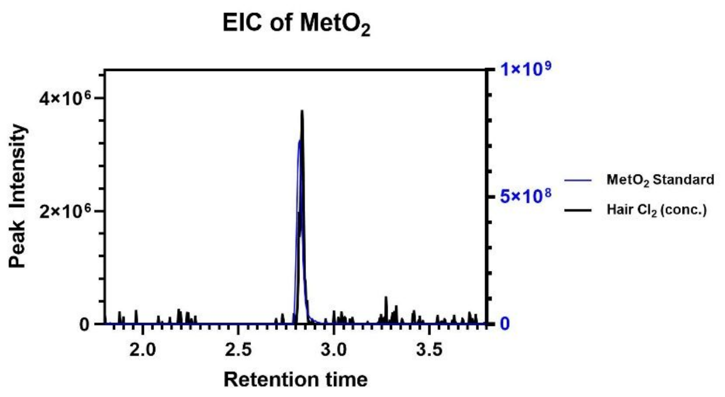

Figure S 7. Comparison of extracted ion chromatogram (EIC) of methionine sulfone ($MetO_2$) obtained from human hair exposed to chlorine conc. (black) and the reference material (blue).

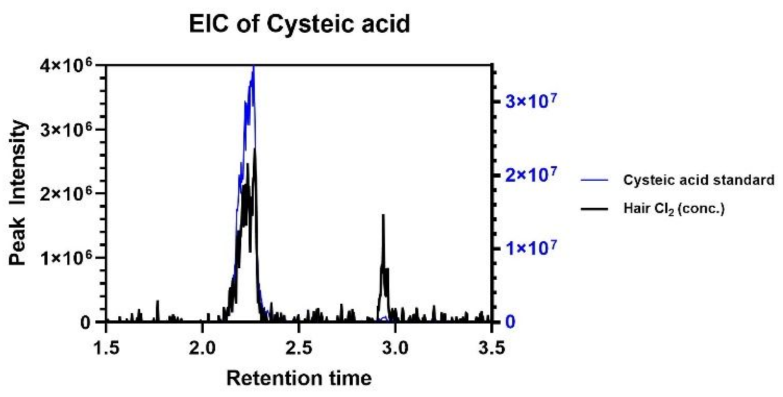

Figure S 8. Comparison of extracted ion chromatogram (EIC) of cysteic acid obtained from human hair exposed to chlorine conc. (black) and the reference material (blue).

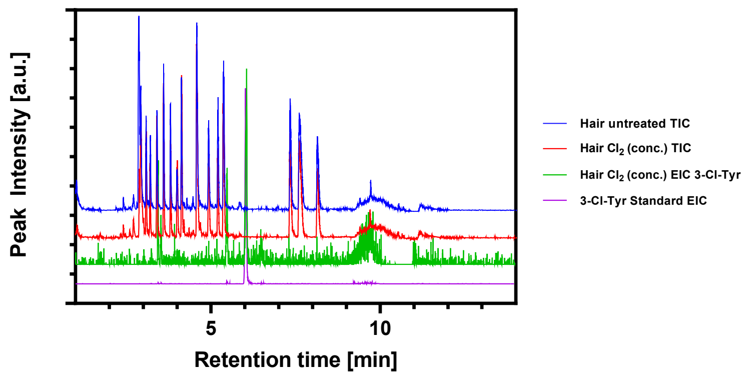

Figure S 9. Total ion chromatogram (TIC) of an untreated hair sample (blue), TIC of Cl_2 exposed hair (red), extracted ion chromatogram (EIC) of 3-Cl-Tyr in the Cl_2 exposed hair sample (green) and the EIC of the 3-Cl-Tyr in the reference material sample (purple). Each chromatogram was normalized to the highest peak.

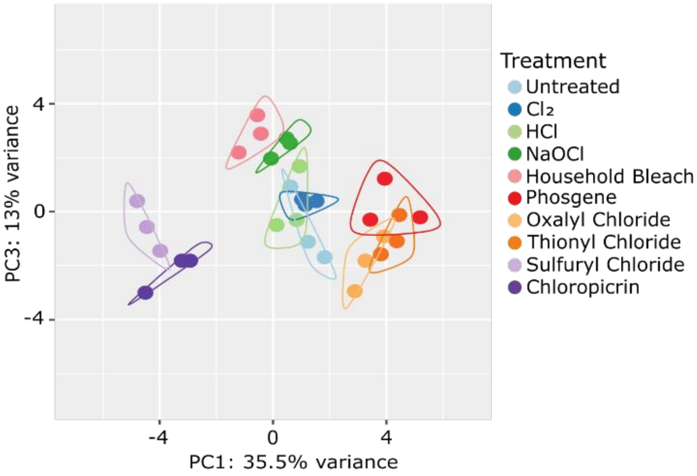

Figure S 10. Separated PCA dimensions PC1 vs. PC3.

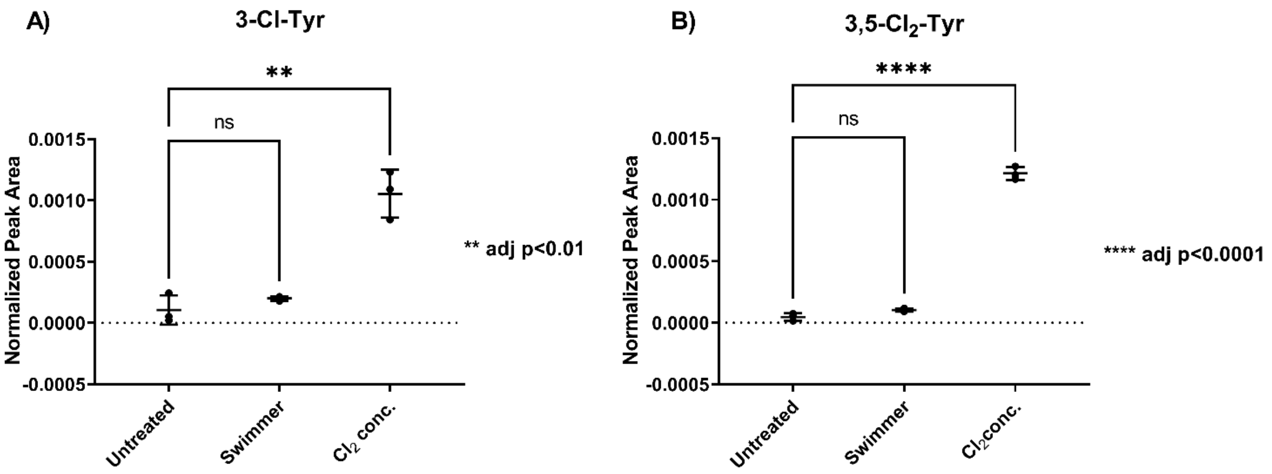

Figure S 11. 3-chlorotyrosine (3-Cl-Tyr) and 3,5-dichlorotyrosine (3,5-Cl_2-Tyr) investigated in untreated hair, hair from a swimmer and Cl_2 conc. exposed hair for comparative purposes. A) 3-Cl-Tyr and B) 3,5-Cl_2-Tyr only observed in significant amounts in hair exposed to concentrated Cl_2. Untreated and Cl_2 exposed hair were measured in biological replicates, whereas the hair samples of the swimmer were measured in technical replicates. Exposure conditions are given in Table S 1. ns: not significant.
